# Performance determinants, running energetics and spatiotemporal gait parameters during a treadmill ultramarathon

**DOI:** 10.1007/s00421-021-04643-2

**Published:** 2021-03-11

**Authors:** Christopher C. F. Howe, Nicola Swann, Owen Spendiff, Anna Kosciuk, Elizabeth K. L. Pummell, Hannah J. Moir

**Affiliations:** School of Life Sciences, Pharmacy and Chemistry, Faculty of Science Engineering and Computing, Kingston University London, Penryhn Road, Kingston upon Thames, KT1 2EE UK

**Keywords:** Endurance, Running, Oxygen cost, Stride frequency, Stride length, Ultra-marathon

## Abstract

**Purpose:**

The objective of this study was to investigate the changes in metabolic variables, running energetics and spatiotemporal gait parameters during an 80.5 km treadmill ultramarathon and establish which key predictive variables best determine ultramarathon performance.

**Methods:**

Twelve participants (9 male and 3 female, age 34 ± 7 years, and maximal oxygen uptake ($\dot{V}$O_2max_) 60.4 ± 5.8 ml·kg^−1^·min^−1^) completed an 80.5 km time trial on a motorised treadmill in the fastest possible time. Metabolic variables: oxygen consumption ($\dot{V}$O_2_), carbon dioxide production ($\dot{V}$CO_2_) and pulmonary ventilation ($\dot{V}$_E_) were measured via indirect calorimetry every 16.1 km at a controlled speed of 8 km·h^−1^ and used to calculate respiratory exchange ratio (RER), the energy cost of running (Cr) and fractional utilisation of $\dot{V}$O_2max_ (*F*). Spatiotemporal gait parameters: stride length (SL) and cadence (SPM) were calculated via tri-axial accelerometery.

**Results:**

Trial completion time was 09:00:18 ± 01:14:07 (hh:mm:ss). There were significant increases in $\dot{V}$O_2_, Cr, *F*, $\dot{V}$_E_ and heart rate (HR) (*p* < 0.01); a significant decrease in RER (*p* < 0.01) and no change in SL and SPM (*p* > 0.05) across the measured timepoints. *F* and Cr accounted for 61% of the variance in elapsed finish time ($R_{{{\text{adj}}}}^{{2}}$ = 0.607, *p* < 0.01).

**Conclusion:**

A treadmill ultramarathon elicits significant changes in metabolic variables, running energetics and spatiotemporal gait parameters. With *F* and Cr explaining 61% of variance in finish time. Therefore, those able to maintain a higher *F*, while adopting strategies to minimise an increase in Cr may be best placed to maximise ultramarathon performance.

## Introduction

Characteristically, ultramarathons are defined as any distance beyond the 42.2 km of the traditional marathon (Hoffman et al. 2010; Knechtle 2012; Krouse et al. 2011; Rüst et al. 2014). Global participation in ultramarathons has increased rapidly over the past 25 years with most individuals taking part in event distances ranging from 50 to 161 km (DUV Ultra Marathon Statistics (2019) Available from: http://statistik.d-u-v.org.). This increase in ultramarathon participation has generated a healthy growth in research around the topic, investigating the complex interaction of several research areas, from physiological, biomechanical, psychological (Howe et al. 2019), and nutritional (Knechtle and Nikolaidis 2018). It has been well documented that a high-level performance in endurance running events up to the marathon, is dependent on a combination of physiological characteristics, such as a high maximal oxygen consumption ($\dot{V}$O_2max_), a large fractional utilisation of $\dot{V}$O_2max_ (*F*), a low energetic cost of running (Cr), as well as optimised muscle activation, recruitment and running biomechanics (Gimenez et al. 2013; Joyner and Coyle 2008; Lazzer et al. 2012; Millet et al. 2011; Saunders et al. 2004; Sjödin and Svedenhag 1985). Cost of running (Cr) is a measure of running economy/efficiency and defined as the energy demand for a given submaximal running speed (Saunders et al. 2004). Running economy is often reported as a stronger predictor of endurance running performance than $\dot{V}$O_2max_ alone (Conley and Krahenbuhl 1980; Daniels 1985), with the most common measure being the oxygen required to cover a given distance (Foster and Lucia 2007; Ingham et al. 2008). However, it has been proposed that Cr is a more accurate predictor of performance than oxygen cost ($\dot{V}$O_2_) (Beck et al. 2018; Fletcher et al. 2009), as $\dot{V}$O_2_ does not account for substrate oxidation rates, with the energy yield per volume of oxygen consumed ~ 7% greater for carbohydrate (CHO) over lipid oxidation (Péronnet and Massicotte 1991). This is a key factor when measuring the energy required during ultramarathons due to the clear shift in substrate oxidation from CHO to lipids (Davies and Thompson 1986; Gimenez et al. 2013; Howe et al. 2018). Traditionally, an increase in Cr is observed in distances up to the marathon (Brueckner et al. 1991); however, the literature regarding changes in Cr during ultramarathons is still open for debate (Vernillo et al. 2017). Previously, it has been demonstrated in a 90 km multi-stage ultramarathon, whereby that $\dot{V}$O_2max,_ *F* and Cr explained 87% of the variance in performance time (Lazzer et al. 2012). Whilst some studies have demonstrated an increase in Cr (Gimenez et al. 2013; Vernillo et al. 2015), others show no change (Balducci et al. 2017; Fusi et al. 2008; Schena et al. 2014; Vernillo et al. 2015), and even a decrease in Cr post-ultramarathon (Vernillo et al. 2014,2016). However, notable differences between studies especially in varying distances and typographies of the ultramarathons studied make it difficult to allow direct comparison. Nevertheless, theories that have been postulated to explain the variation in reported Cr in ultramarathons include, a decline in the functional capacity of the respiratory system (Wuthrich et al. 2015), with some studies showing an increase of ~ 18% in pulmonary ventilation ($\dot{V}$_E_) (Millet et al. 2000; Vernillo et al. 2014), however, other studies have shown a decrease of between 3–10% in $\dot{V}$_E_ post-ultramarathon (Gimenez et al. 2013; Lazzer et al. 2015; Schena et al. 2014), in distances ranging between 60 and 330 km. Alterations in neuromuscular function are also evident, due to the fatiguing nature and muscle damage accrued during ultramarathons (Knechtle and Nikolaidis 2018; Nieman et al. 2005), which requires increased neural input to the working muscles to maintain the same force output, resulting in an increase in oxygen demand and therefore increase in Cr (Bigland-Ritchie and Woods 1974). This may lead to changes in muscle activation, through the recruitment of less efficient type II muscle fibres causing changes in biomechanical parameters (Degache et al. 2016; Morin et al. 2011b), specifically changes in spatiotemporal gait parameters, such as increased stride frequency [steps per minute (spm)] and reduced stride length (SL) (Cavanagh and Kram 1989; Cavanagh and Williams 1982; Moore 2016). While it is possible to extrapolate some data generated from short endurance running research (Millet et al. 2011), due to the extreme and demanding nature of ultramarathon running this is not always possible (Burns et al. 2019; Gimenez et al. 2013; Millet et al. 2002; Scheer et al. 2018) due to event specific inter- and intra-variability and range of training status and expertise level of participants. The differing methodological approaches employed in ultramarathon research make direct comparisons of data challenging, both between events and within an individual event (Vernillo et al. 2017).

Ultramarathons can be single- or multi-stage, have varying terrain across and within events (treadmill, track, desert and mountainous etc.) along with environmental factors (temperature and altitude), all of which elicit different metabolic responses (Burns et al. 2019; Gimenez et al. 2013; Lazzer et al. 2014; Millet et al. 2002; Scheer et al. 2018). However, due to the remote nature of many ultramarathons and the logistical challenges of collecting data, the majority of studies have only been able to collect pre- and post-ultramarathon measurements (Fusi et al. 2008; Lazzer et al. 2012; Vernillo et al. 2014), with few investigating the adjustments and change in energetic and metabolic variables during an ultramarathon (Gimenez et al. 2013; Millet et al. 2011). The use of treadmill running, whilst different to ecologically valid ultramarathon races/events, due to the lack of competition and effect of environmental factors, provides a useful tool to collect and facilitate real-time ‘in-event’ data collection (Morin and Sève 2011).

Therefore, the aim of this study was to investigate the changes and adjustments in running energetics and metabolic variables and spatiotemporal gait parameters during an 80.5 km treadmill ultramarathon and establish which key predictive variables best determine ultramarathon performance. It was first hypothesised that completing a treadmill ultramarathon would elicit an increase in Cr and SPM with a decrease in SL. Second, it was hypothesised that a combination of a high $\dot{V}$O_2max_, large *F* and low Cr would all contribute to overall performance.

### Methodology

#### Experimental design and participants

Fourteen endurance runners with a minimum of 3 years running experience and no known injuries were initially recruited via social media and ‘word of mouth’ through the local ultramarathon community, but due to the nature of the study protocol, only twelve participants (9 male and 3 female) completed the 80.5 km distance. The study was approved by the Faculty of Science, Engineering and Computing Ethics Committee at Kingston University London and all volunteer participants provided written informed consent to participate. All procedures were conducted in accordance with the Declaration of Helsinki. Participant anthropometric and physiological characteristics are reported in Table 1. All participants were required to visit the human performance laboratory at Kingston University London on two occasions (Visit 1: baseline measurements and maximal discontinuous incremental $\dot{V}$O_2max_ test. Visit 2: 80.5 km treadmill ultramarathon), with visits separated by at least one week but no longer than three weeks apart.

**Table 1:** Participant characteristics and maximal discontinuous incremental $\dot{V}$O_2max_ test (*n* = 12)

Variable	Mean ± SD
Age (years)	34 ± 7
Stature (cm)	173.7 ± 7.3
Body mass (kg)	68.4 ± 7.4
BMI (kg/m^2)^	22.6 ± 1.9
Body fat (%)	15.1 ± 5.1
Ave weekly training distance (km)	74 ± 27
$\dot{V}$O_2max_ (ml·min^−1^)	4168 ± 482
$\dot{V}$O_2max_ (ml·kg^−1^·min^−1^)	60.4 ± 5.8
HR_max_ (bpm)	187 ± 13
${\rm{V}}_{\dot{V}{\text{O}}_{2\max }}$ (km·h^−1^)	17.3 ± 1.8

*BMI* body mass index, $\dot{V}$*O*_*2max*_ maximal oxygen uptake capacity, *HR*_*max*_ maximum recorded heart rate, ${V}_{\dot{V}{\text{O}}_{2\max }}$ maximum treadmill speed obtained

### Instruments

Metabolic variables ($\dot{V}$O_2_, $\dot{V}$CO_2_ and $\dot{V}$_E_) were measured via indirect calorimetry using the Oxycon Pro metabolic cart (Vyaire, UK), which was calibrated according to manufacturer guidelines prior to every measurement. Spatiotemporal parameters were calculated from raw tri-axial accelerometer data (GT3X + , ActiGraph, LLC, Fort Walton Beach, FL) which was initialised using the device software (Actilife 5, ActiGraph, LLC, Fort Walton Beach, FL) and set to collect data at a sampling rate of 100 Hz. Raw accelerometer data were analysed for peak vertical accelerations at the hip via a custom MATLAB (The MathWorks, Inc., Natick, MA, USA) script to calculate spatiotemporal gait parameters; stride time, cadence (strides per minute (SPM)) and stride length (SL). Tri-axial accelerometers have previously demonstrated suitable levels of agreement in identifying spatiotemporal gait parameters when compared to an infrared camera laboratory-based system (Lee et al. 2010). The GT3X + was placed inside a neoprene pouch attached to an elasticised waistband which was then attached on the participant’s dominant hip on the mid-axillary line for the duration of the 80.5 km trial. HR was measured continuously throughout all trials using a HR strap fitted around the participant’s chest and the data transmitted via telemetry (Polar Electro Oy, Kempele Finland).

### Baseline measurements and maximal discontinuous incremental $\dot{V}$O_2max_ test

Participant’s anthropometrics and body composition were measured at baseline. Stature (cm) was measured using a floor stadiometer (Holtain Ltd., Dyfed, Wales) and body mass (kg) using electronic scales (Seca, Vogel and Halke, Germany). Body fat % was estimated using air-displacement plethysmography (Bod Pod Cosmed, Rome, Italy). Participant’s maximal oxygen uptake ($\dot{V}$O_2max_), performed on a motorised treadmill (H/P/Cosmos, Nussdorf-Traunstein, Germany), was assessed using a maximal discontinuous incremental protocol to volitional exhaustion. The treadmill (H/P/Cosmos Venus) was set at an incline of 1% (Jones and Doust 1996) and an initial speed of 10 km·h^−1^ for a period of 6 min, followed by a 1 min standing rest for the collection of a capillary blood sample (Biosen C-Line Sport EKF diagnostic, Germany). The treadmill speed was then increased by 1.5 km·h^−1^ every 3 min followed by a 1 min standing rest to allow for capillary blood collection to measure blood lactate (mmol·L^−1^), this was repeated until volitional exhaustion. The discontinuous protocol was designed to allow comparison to previous studies (Gimenez et al. 2013) as well as to allow participants to run at the higher speeds for a sufficient period of time to provide steady-state values.

### 80.5 km treadmill ultramarathon

Visit two comprised an 80.5 km treadmill ultramarathon. To replicate a race of this distance, participants were instructed to complete the distance in the fastest possible time, with the exception of a 3 min period at a control speed of 8 km·h^−1^, beginning 10 min after commencing the trial (0 km) and then at every 16.1 km interval thereafter. Throughout each 3 min period, respiratory variables were measured via indirect calorimetry (Oxycon Pro, Vyaire, UK). The last minute of each 3 min period was used for analysis once steady state was achieved. Steady state was checked using the method outlined by De Ruiter et al. (2014), by calculating the slope of a linear regression line fitted through the $\dot{V}$O_2_ data of the third min, where steady state was confirmed if zero slope fell within the individual 95% confidence intervals. The use of a control speed enabled both inter- and intra-direct comparison by removing self-selected velocity as a confounding factor. The 8 km·h^−1^ speed was selected to allow comparison to previous research as well as this speed having been suggested to best represent an average pace for this type of activity (Gimenez et al. 2013; Millet et al. 2011). The accelerometer and HR data were time-matched with the respiratory variables for subsequent analysis. Food and drink were available *ad libitum* during the entire duration of the trial and self-selected according to the participant’s preference, to replicate their habitual ultramarathon practices. All nutritional intake was recorded and analysed through nutritional analysis software (Dietplan 6 Software, Horsham, U.K.). Further details are reported in previously published work (Howe et al. 2019).

### Energy cost of running

The last minute of the 3 min collection period when steady state was observed was averaged and used to calculate Cr. Cr is expressed as joules per kilogram per meter (J·kg^−1^·m^−1^), using the associated caloric equivalent of oxygen (kJ·L^−1^) depending on respiratory exchange ratio (RER) (Péronnet and Massicotte 1991) using the following equation.

Cr (J·kg^−1^·m^−1^) =  $\dot{V}$O_2_ (L·min^−1^) _•_ caloric equivalent of O_2_ (kJ·L^−1^)/body mass (kg)/speed (m·min^−1^).

Due to the issues around baseline subtraction of resting metabolic rate (RMR), it cannot be confirmed that RMR is maintained at the same rate during running compared to rest (Fletcher et al. 2009; Stainsby and Barclay 1970), the decision was made not to subtract RMR. All respiratory variables that are expressed relative to body mass are adjusted to the actual body mass loss as measured at every 16.1 km split throughout the 80.5 km trial.

### Statistical analysis

All data were assessed for normality via the Shapiro–Wilk normality test. Differences between 16.1 km intervals for all variables measured were analysed by one-way repeated measures analysis of variance (ANOVA) with Bonferroni post hoc*.* Effect sizes were calculated using partial ETA^2^ (ηp^2^) where 0.01 = small; 0.06 = medium; and 0.14 = large effect (Field 2018). Pearson’s correlations (*r*) were used to examine for significant relationships between potential determinants of ultramarathon performance and elapsed finished time for the 80.5 km trial. Those determinants found to have a significant linear relationship with performance time (*p* ≤ 0.05), were entered into a multiple linear regression to assess the relative contribution to performance. Delta change (Δ) of selected variables was calculated from the 80.5 km distance measurement minus the start 0 km distance measurement and expressed as a percentage of start 0 km distance measurement (%Δ). All data were analysed using IBM SPSS Statistics 24 (SPSS Inc., Chicago, IL, USA) and presented as mean ± SD and the alpha level was set at *p* ≤ 0.05.

## Results

### Performance measures

The average elapsed time and average moving time to complete the 80.5 km trial were 540 ± 74 and 500 ± 64 min, respectively (9.81 ± 1.31 and 9.1 ± 1.28 km·h^−1^, respectively). The mean speed over the 80.5 km TT expressed as a percentage of speed associated with $\dot{V}$O_2max_ equals 57.0 ± 6.0%${\rm{V}}_{\dot{V}{\text{O}}_{2\max }}$ when considering moving time on the treadmill. When expressed as elapsed time, including comfort breaks etc., akin to a race and time taken to perform measurements, the mean *F* equals 66.5 ± 3.0%$\dot{V}$O_2max_ and mean elapsed %${\rm{V}}_{\dot{V}{\text{O}}_{2\max }}$ equals 52.8 ± 6.7%.

### Body mass and nutritional intake

There was an overall significant decrease in participant body mass over the 80.5 km TT (*F* (1.28, 14.08) = 68.97, *p* < 0.01, ηp^2^ = 0.862) with a significant decrease identified at all 16.1 km splits (*p* < 0.05), apart from between 48.3 and 64.4 km (*p* = 0.097). Pre- to post-trial exercise-induced body mass loss was 2.6 ± 0.97 kg (*p* < 0.001), which equated to a percentage loss of 3.9 ± 1.32%. Total mean energy intake during the 80.5 km trial was 6.68 ± 2.35 mega joules and 1588 ± 553 kilocalories. Macronutrient intake was 37.18 ± 12.6.0 g·h^−1^ CHO, 2.64 ± 2.0 g·h^−1^ fat and 8.33 ± 1.1 g·h^−1^ protein. Further details can be seen in previously published work (Howe et al. 2019).

### O_2_ cost, Cr and *F*

**Fig. 1:**
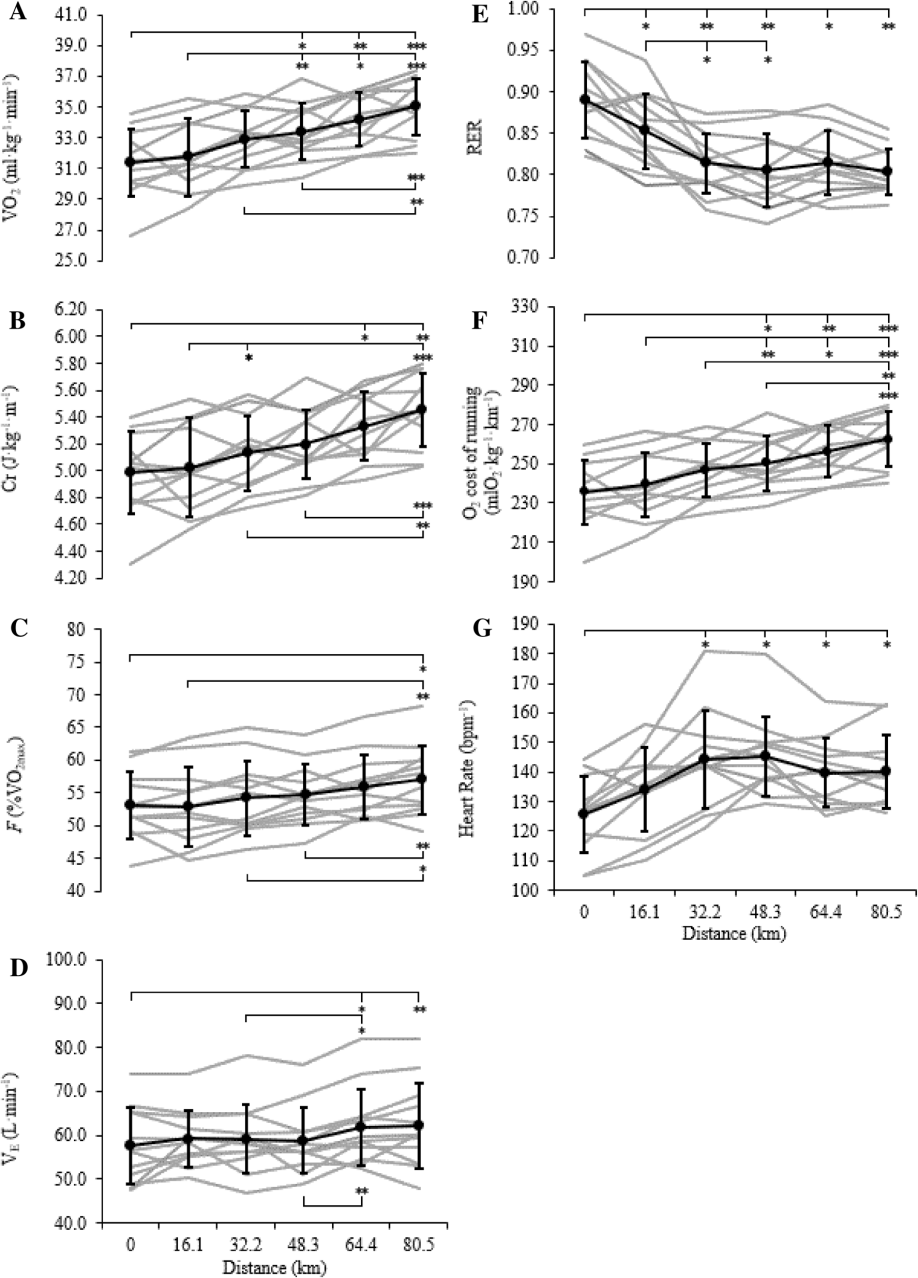
Change in **a** $\dot{V}$O_2_ (ml·kg^−1^·min^−1^), **b** energy cost of running (Cr, J·kg^−1^·m^−1^), **c** fractional utilisation of $\dot{V}$O_2max_ (*F*, %$\dot{V}$O_2max_), **d** minute ventilation ($\dot{V}$_E_) **e** respiratory exchange ratio (RER), **f** O_2_ cost of running (mlO_2_·kg^−1^·km^−1^) and **g** heart rate (bpm^−1^) measured at 16.1 km intervals at the control speed. Solid black line: mean ± SD. Significance set at *p < 0.05, **p < 0.01, ***p < 0.001. *Grey lines* individual participant responses

A significant increase in relative $\dot{V}$O_2_ (ml·kg^−1^·min^−1^) adjusted for the reduction in body mass throughout the 80.5 km TT at the control speed was observed (*F* (5, 55) = 21.33, *p* < 0.01, ηp^2^ = 0.66). Post hoc analysis identified a significant increase from the start of the TT to distance points of 48.3, 64.4 and 80.5 km (*p* < 0.05) and this continued to increase up to the completion of the 80.5 km ultramarathon (*p* < 0.01) (Fig. 1a). A significant increase was in the O_2_ cost of running at the control speed when expressed as mlO_2_·kg^−1^·km^−1^ (*F* (5, 55) = 21.18, *p* < 0.001, ηp^2^ = 0.658). Post hoc analysis identified a significant increase in the O_2_ cost between the start and 48.3, 64.4 and 80.5 km (*p* < 0.05, *p* < 0.01 and *p* < 0.001, respectively) (Fig. 1f). There was a significant increase in Cr when RER was taken into account (*F* (5, 55) = 15.65, *p* < 0.001, ηp^2^ = 0.59). Post hoc analysis identified a significant increase from the start to the 64.4 and 80.5 km distances (*p* < 0.05 and *p* < 0.01, respectively), and from the 16.1 km distance to 32.2 and 80.5 km (*p* < 0.05 and *p* < 0.01, respectively), between 32.2 and 80.5 km (*p* < 0.01) and between 48.3 and 80.5 km (*p* < 0.01) (Fig. 1b). A significant increase *F* was observed (*F* (5, 55) = 10.95, *p* < 0.01, ηp^2^ = 0.5) at the control speed, with post hoc analysis showing a significant increase from the start to finish and the 16.1, 32.2 and 48.3 km distance points to the finish (*p* < 0.05) (Fig. 1c).

### RER, $\dot{V}$_E,_ and HR

A significant decrease in RER was observed at the control speed (*F* (1.96, 2.54) = 17.21, *p* < 0.01, ηp^2^ = 0.61), with post hoc analysis identifying a significant decrease from the start (0 km) and all 16.1 km intervals, as well as between 16.1 and 32.2 and 48.3 km splits (*p* < 0.05), after which RER plateaus (Fig. 1e). There was a significant overall increase in $\dot{V}$_E_ at the control speed of 8 km·h^−1^ (*F* (5, 55) = 3.64, *p* < 0.01, ηp^2^ = 0.5), with post hoc analysis showing a significant increase from the start to 64.4 and 80.5 km (*p* < 0.05 and *p* < 0.01, respectively). There was also a significant increase between 32.2 and 64.4 km (*p* < 0.05) and between 48.3 and 64.4 km (*p* < 0.01) (Fig. 1d). At the control speed, there was an observed mean HR of 75 ± 4% of HR_max_. HR significantly increased over the duration of the 80.5 km trial at the control speed (*F* (2.5, 27.9) = 10.31, *p* < 0.01, ηp^2^ = 0.48). Post hoc analysis identified a significant increase from the start (0 km) to 32.2, 48.3, 64.4 and 80.5 km distances (*p* < 0.05) after which a plateau is evident (Fig. 1g).

### Spatiotemporal gait parameters

**Fig. 2:**
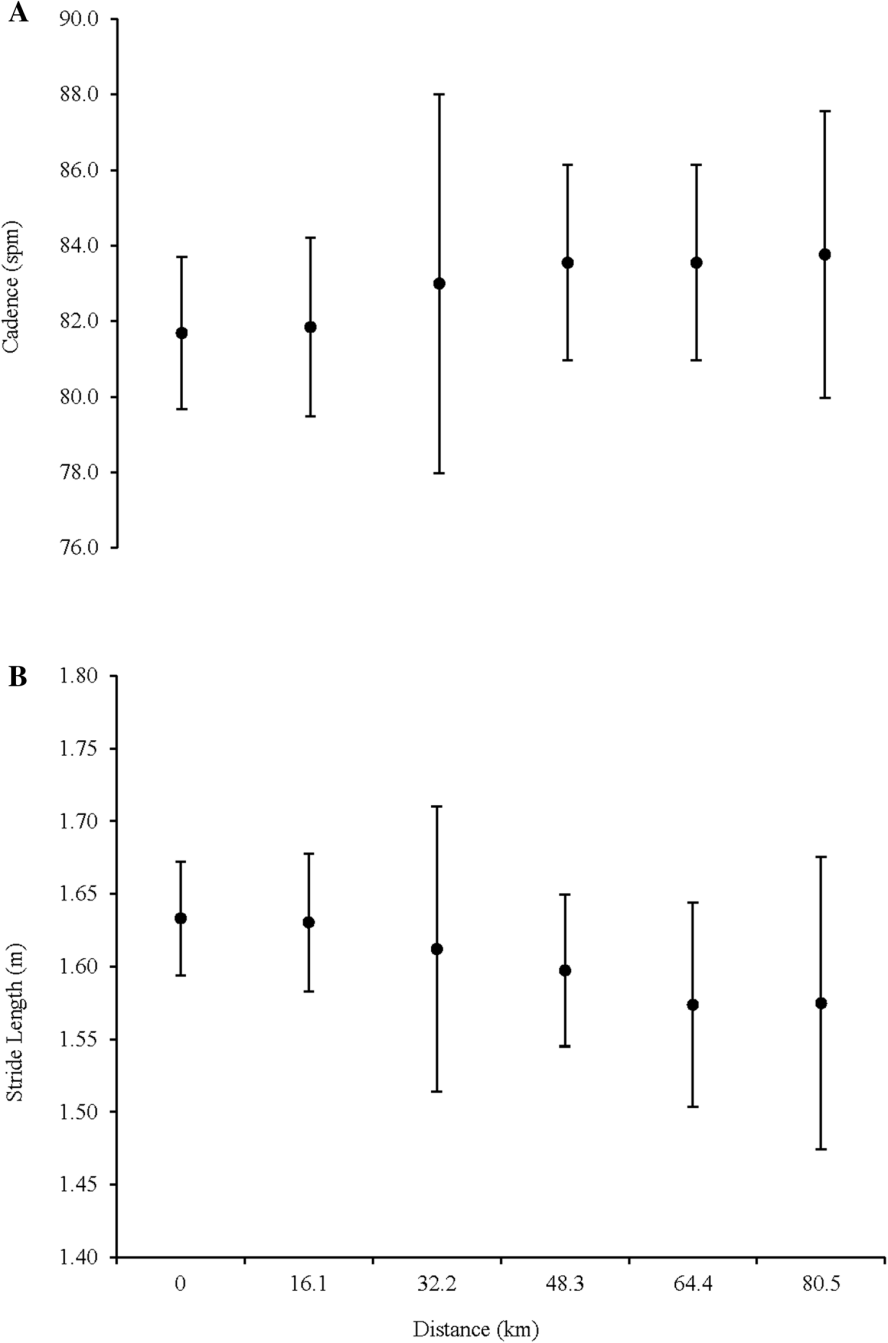
Change in spatiotemporal parameters **a** Cadence (SPM) **b** stride length (m) measured at 16.1 km intervals at the control speed of 8 km·h^−1^ Data is presented as means ± SD

There was no significant change in the spatiotemporal parameters measured across all 16.1 km measurement splits at the control speed of 8 km·h^−1^; SPM (*F* (2.02, 22.3) = 1.76, *p* = 0.195, ηp^2^ = 0.14, observed power = 0.33), and SL (*F* (2.4, 26.3) = 2.28, *p* = 0.11, ηp^2^ = 0.17, observed power = 0.46) at the control speed (Fig. 2a, b).

### Pearson’s correlations and multiple linear regression

**Fig. 3:**
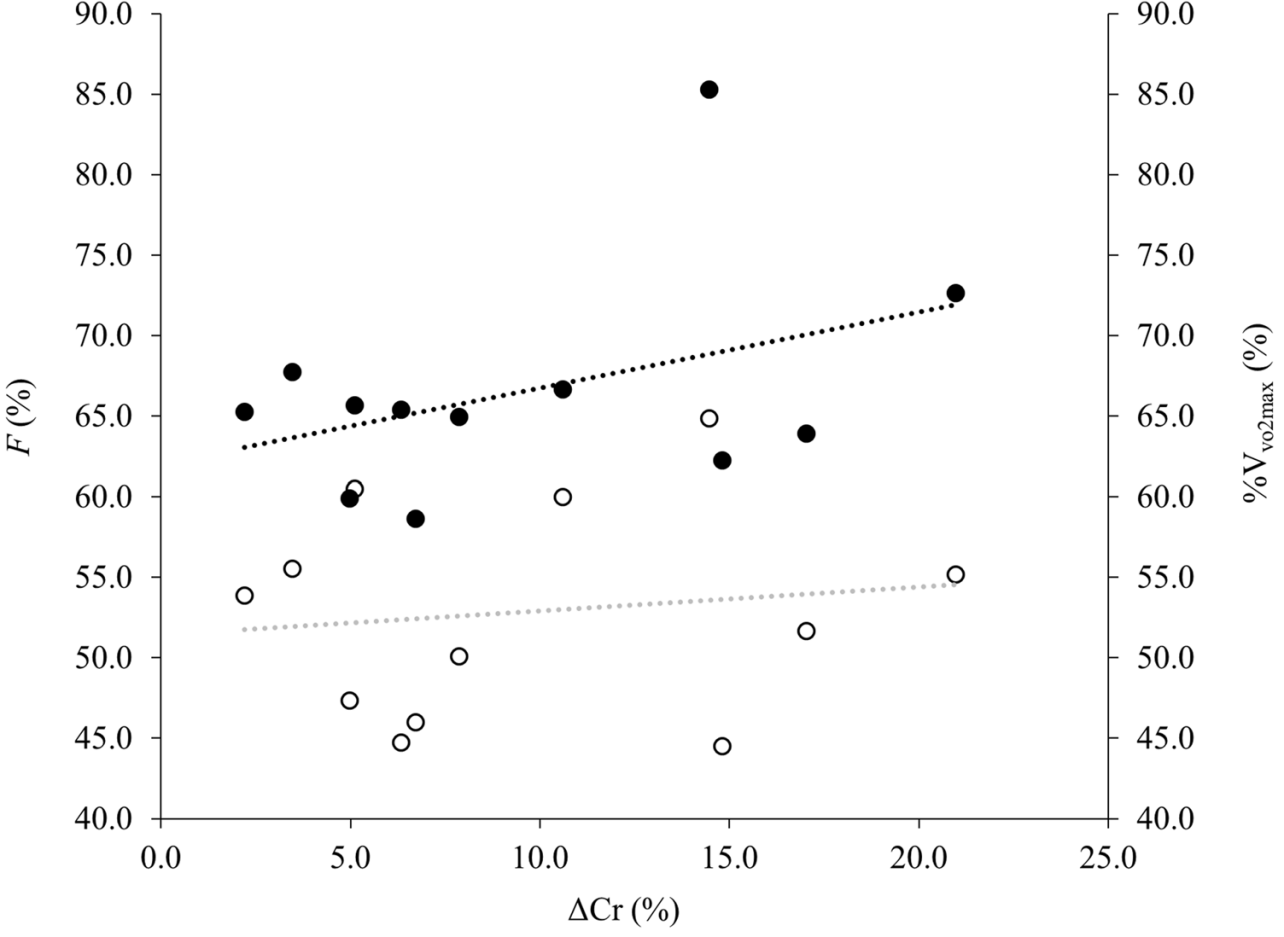
Relationship between the mean fractional utilistation of $\dot{V}$O_2max_ as a percentage of $\dot{V}$O_2max_ (*F*) (closed circles and black dashed line) and the percentage of $\dot{V}{\text{O}}_{2\max }$ (open circles and grey dashed line) sustained during the 80.5 km trial compared to change in energy cost of running at the control speed from start to finish of the 80.5 km trial ΔCr (%)

**Fig. 4:**
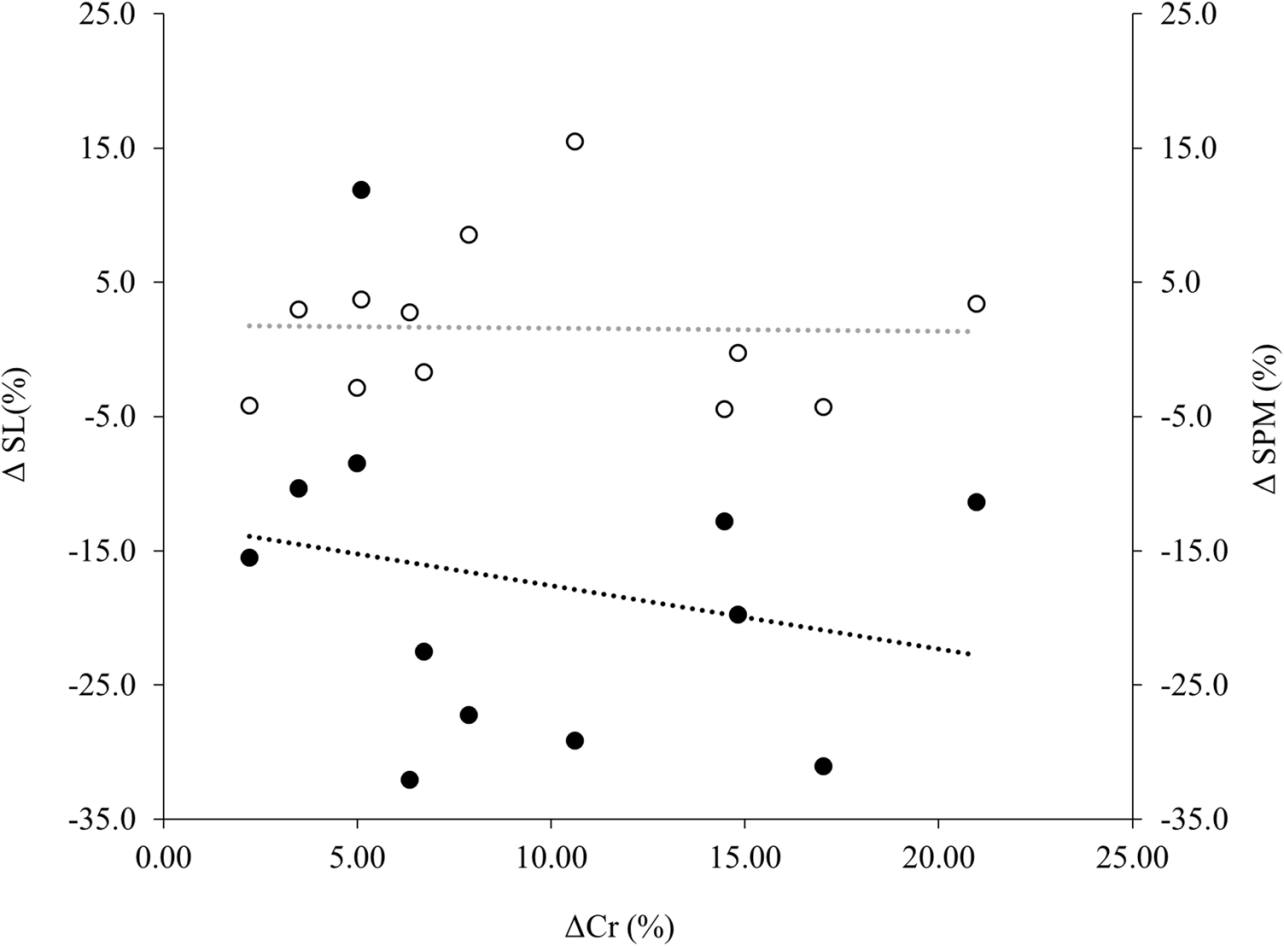
Relationship between the percentage change in SL [ΔSL (%)] (closed circles and black dashed line) and the percentage change in running cadence [Δcadence (%)] (open circles and grey dashed line) during the 80.5 km trial compared to change in energy cost of running at the control speed from start to finish of the 80.5 km trial ΔCr (%)

Pearson’s correlations were performed between elapsed finish time and endurance performance determinants ($\dot{V}$O_2max_, *F*, and Cr); where *F* and Cr were measured for 3 min periods at 16.1 km intervals at a self-selected speed and averaged to give a mean *F* and Cr over the 80.5 km ultramarathon. There were significant linear relationships between both *F* and Cr and elapsed finish time (*r* = 0.63, *p* = 0.015 and *r* = 0.61, *p* = 0.018, respectively); however, there was no relationship between $\dot{V}$O_2max_ of participants and their performance time (*r* = − 0.23, *p* = 0.24). Due to absence of a relationship between $\dot{V}$O_2max_ and finish time only, *F* and Cr were entered into the multiple linear regressions, which identified a significant relationship between these determinants and finish time (*F* (2, 9) = 9.5, *p* = 0.06, *R*^2^ = 0.68, $R_{{{\text{adj}}}}^{2}$ = 0.61). Therefore, in the study cohort, 61% of the variance in elapsed finish time can be explained by a higher sustained *F* and lower Cr throughout the 80.5 km ultramarathon. There were no significant correlations between mean *F* and ${\rm{V}}_{\dot{V}{\text{O}}_{2\max }}$ (%) sustained throughout the 80.5 km trial compared to ΔCr (%) using the control speed (*r* = 0.14; *p* = 0.68 and *r* = 0.41; *p* = 0.19, respectively; Fig. 3). As well as no significant correlation between ΔSL (%) and Δcadence (%) at self-selected running speed when compared to ΔCr (%) (*r* = − 0.23; *p* = 0.48 and *r* = 0.02; *p* = 0.95, respectively; Fig. 4). There was a significant positive correlation between Cr and the oxygen cost of running (mlO_2_·kg^−1^·km^−1^), (*r* = 0.99, *p* < 0.001).

## Discussion

This study aimed to determine the variation in metabolic variables, running energetics and spatiotemporal gait parameters during an 80.5 km treadmill ultramarathon and identify the level to which key variables ($\dot{V}$O_2max_, *F* and Cr) influence overall performance. The use of a control speed of 8 km·h^−1^ with 16.1 km interval analysis allowed for precise intra- and inter-observation of changes in the measured parameters throughout the duration of an ultramarathon treadmill run. The key findings illustrated a significant increase in $\dot{V}$O_2_, Cr, *F*, $\dot{V}$_E_ and HR and a significant decrease in RER, no change in spatiotemporal gait parameters of SL and cadence. When investigating performance determinants, the results indicated that *F* and Cr explained 61% of the variance in performance time, whilst $\dot{V}$O_2max_ did not contribute to or correlate with overall performance time. However, it must be noted that due to the homogeneity of the participants ($\dot{V}$O_2max:_ 60.4 ± 5.8 ml·kg^−1^·min^−1^), $\dot{V}$O_2max_ may prove to contribute additionally to overall performance in a more heterogenous group.

To allow comparison to a real-life race scenario, participants freely selected their running speed throughout the ultramarathon, bar a 3 min period at the control speed of 8 km·h^−1^ every 16.1 km from the start. Elapsed mean running speeds were between 7.6 and 11.4 km·h^−1^, with a mean speed decrease of 2.2 ± 0.9 km·h^−1^ observed between the first and second half of the 80.5 km distance, the variation of which illustrates the requirement to use a consistent controlled speed for analysis.

To date, evidence relating to oxygen cost and Cr in ultramarathons has been conflicting (Balducci et al. 2017; Fusi et al. 2008; Gimenez et al. 2013; Schena et al. 2014; Vernillo et al. 2014,2015,2016), owing mainly to the variance in event distance/duration as well as terrain and environmental conditions. Therefore, using a laboratory-based study design enabled the standardisation and control of conditions. Here, we observed an increase in both the $\dot{V}$O_2_ and Cr (Fig. 1a, b) at the control speed. Whilst the increase in $\dot{V}$O_2_ and Cr, both follow similar upward trends throughout the 80.5 km trial, the significant decrease in RER from the start of the trial to the 32 km measurement distance marginally reduces the increase in Cr compared to V̇O_2_ alone, through the inclusion of substrate utilisation (RER). The shift to fatty acid oxidation is well documented in the literature (Brueckner et al. 1991; Costill 1970; Howe et al. 2018), and this corresponds with the related increase in Cr occurring in the first 32 km [02:45:03 ± 00:25:58 (hrs:mins:sec)] before the observed plateau (Fig. 1e). The shift to fatty acid oxidation has also been confirmed using a novel untargeted metabolomic approach in the same cohort, which was demonstrated by a marked increase in peroxisomal metabolism providing acetyl carnitines for export to mitochondria in the muscles (Howe et al. 2018). Whilst the findings in this study are in agreement with previous literature (Gimenez et al. 2013; Lazzer et al. 2012), the plateau in RER occurred much earlier at ~ 3 h compared to a significant decrease in RER up to the 8 h time point before plateauing during a 24 h treadmill ultramarathon at the same control speed of 8 km·h^−1^ (Gimenez et al. 2013). The difference observed here may partly be explained by the shorter distance (80.5 km vs. mean 149.2 ± 15.7 km) and higher *F* sustained by the participants in this study (52.8 ± 6.7% vs. 39 ± 4%), due to an accelerated glycogen depletion and therefore a more rapid shift to fatty acid oxidation with a *F*. In addition, participants were asked to cover the 80.5 km in the ‘fastest possible time’ whereas in the study of Gimenez et al. (2013), participants were instructed to achieve the ‘greatest distance’ in a given time period (24 h), suggesting individual pacing and perception of effort required may be a factor in the difference in RER plateaus. The lower trained status of the ultramarathon runners in the study conducted by Gimenez and colleagues (2013) compared to this study ($\dot{V}$O_2max_ 52.0 ± 6.3 vs. 60.4 ± 5.8 ml·kg^−1^·min^−1^, respectively) may also explain the difference, with more trained individuals having a greater ability to oxidise fat (Purdom et al. 2018). When looking at self-selected running speed, there is no change in $\dot{V}$O_2_, with a significant decrease in *F* and significant increase in Cr over the course of the 80.5 km ultramarathon. Both the $\dot{V}$O_2_ and *F* data can be explained by the decrease in running speed through the trial, whilst the increase in Cr despite the decrease in running speed is part explained by the significant decrease in RER mirroring that observed at the control speed. However, it must be recognised as a potential limitation that the variance in individual self-selected running speeds chosen by the participants could influence the data collected at the control speed, especially as control speed was much lower for some participants compared to self-selected running speeds, but less so later in the trial when self-selected and control speeds were similar. To mitigate for this, sufficient time allowed ‘steady-state’ to be reached at the control speed when measuring metabolic variables ($\dot{V}$O_2_, $\dot{V}$CO_2_ and $\dot{V}$_E_).

The increase in Cr during ultra-endurance exercise is still not fully understood (Gimenez et al. 2013), although several suggestions have been proposed. For example, an increase in $\dot{V}$_E_ and its association with higher respiratory frequency has been suggested in shorter duration events (Davies and Thompson 1986; Millet et al. 2000), which is supported in our findings, such that a significant increase in $\dot{V}$_E_ was evident between the start and end of the 80.5 km ultramarathon (Fig. 1d). Interestingly, this was not observed in a 24 h treadmill run where $\dot{V}$_E_ did not significantly change over the course of the exercise (Gimenez et al. 2013), perhaps due to the difference in mean running speed between the studies 7.9 ± 1.0 km·h^−1^ for the 24 h treadmill vs. 10.3 ± 1.3 km·h^−1^. Caillaud and colleagues (1995), proposed the increase in Cr is due to the increase in $\dot{V}$_E_ attributed to the change in O_2_ diffusion across the alveolar-capillary membrane, however, this was not measured in the current study and therefore warrants further investigation in prolonged ultra-endurance activities. Further explanations for an increase in Cr may be peripheral muscular alterations (Gimenez et al. 2013), a reduction in mitochondrial efficiency (Fernström et al. 2007), and changes in muscle activation, efficiency, or in biomechanical parameters, such as stride frequency (Morin et al. 2011a). However, although there was a slight upward trend in cadence and slight downward trend in SL, no statistically significant changes were observed in these spatiotemporal parameters at the control speed (Fig. 2). This is likely to be the result of changing the speed resulting in a forced alteration of running gait. This adaptation in control speed may have required in some instance a reduction/change in preferred SL and frequency. While sufficient time allowed ‘steady state’ to be reached at the control speed for metabolic variables, it may not have been long enough for the stabilisation of gait patterns. This may therefore have resulted in a wider range of speed alterations for some, resulting in greater gait variability. Future investigations should consider the inclusion of ground contact time and duty cycle data to estimate force generation capacity of the leg and increases in metabolic energy expenditure to further explore the mechanisms involved in the increase in Cr (Beck et al. 2020; Taylor and Kram 1990).

It has previously been demonstrated that an increase in SPM coupled with a decrease in SL occurs over the duration of an ultramarathon to adopt a “smoother” or “safer” running technique (Vernillo et al. 2019). This was not observed in the currently study, potentially due to the differences between over-ground and treadmill running and no natural variation in terrain, and the reduced braking and propulsive forces experienced and more ordered control in gait whilst running on a treadmill (Lindsay et al. 2014). However, changes are potentially explained by the fatiguing nature of an ultramarathon, with increased muscle damage and inflammation along with reduction in muscle force generation over the gait cycle, resulting in a safer running technique being adopted to limit further damage, leading to a greater Cr due to the newly adopted cadence and stride length (Morin et al. 2011; Vernillo et al. 2019). Furthermore, it has been demonstrated that participants who maintained the highest %${\rm{V}}_{\dot{V}{\text{O}}_{2\max }}$ during a 24 h treadmill ultramarathon also displayed the largest deterioration in Cr, which raises the question that a lower Cr is of less significance in ultramarathon performance (Gimenez et al. 2013). However, there was no correlation observed between those participants that maintained the highest *F* (%) and %${\rm{V}}_{\dot{V}{\text{O}}_{2\max }}$ with the largest increases in Cr (Fig. 3), nor between those who had the great change in SL and cadence and ΔCr (Fig. 4). This therefore indicates that $\dot{V}$O_2max_ did not contribute to overall performance, with *F* and Cr explaining 61% of variance in finish time. It has previously been reported that as distance increases, $\dot{V}$O_2max_ becomes of less importance (Davies and Thompson 1979; Sjödin and Svedenhag 1985), however, having a high $\dot{V}$O_2max_ enables a higher submaximal speed or *F* to be maintained (Millet et al. 2011), which may indicate why *F* demonstrated great predictive power of performance in the current study. The current findings contradict those of Lazzer et al. (2012) where 87% of performance time was attributed to $\dot{V}$O_2max_, *F* and Cr in a 3 days ultra-endurance running event, however, in their study, HR was used as a estimation of *F*, which may lead to small but systematic errors, compared to indirect calorimetry that was used in the current study. To the authors’ knowledge, the current study is one of very few studies to directly measure these variables during an ultramarathon.

While no changes were observed at the control speed, there was a significant reduction in SL at self-selected speeds over the course of the ultramarathon, which is a function of a reduction in running speed but again this indicates suggests that runners naturally selected a cadence and SL that is optimal or very near to being economically optimal (Moore et al. 2012; Moore 2016; Williams and Cavanagh 1987). Indeed, this difference was likely a result of the controlled speed producing forced alterations in running gait. There was no change in running cadence during self-selected running speeds, and a potential explanation for the increase in Cr with no significant change in spatiotemporal parameters, maybe the change in mechanical properties of the Achilles tendon, such as demonstrated following a 90 min submaximal run which showed a small but significant increase in Cr, by increased muscle fascicle shortening (Fletcher and MacIntosh 2018). Though, it must be noted, that the alternations in running speed between self-selected and control, may have also contributed to this. As mechanical properties were not measured, it should be taken into consideration when designing future investigation into the mechanisms behind changes in Cr in ultramarathon distance events. It is important to acknowledge the benefits and constraints of using a treadmill to analysis running performance. In a recent systematic review and meta-analysis, there was no difference observed between over-ground running and motorised treadmill running for submaximal $\dot{V}$O_2_, heart rate and perceived effort; however, preferred submaximal running speeds were slower and blood lactate concentrations were lower whilst running on a treadmill compared to over-ground (Miller et al. 2019). Whilst, significant differences in ground reaction forces and kinematic variables were observed between over-ground and graded treadmill running, the differences were small and the researchers believe that variables measured on a treadmill may be applicable to over-ground running (Firminger et al. 2018). This is further supported by a meta-analysis comparing biomechanical measures between over-ground and treadmill running, where spatiotemporal, kinematic, kinetic, muscle activity, and muscle–tendon measures were widely comparable (Van Hooren et al. 2019). Therefore, the authors feel confident that the data presented have ecological validity to external ultramarathon events with minimal course elevation.

## Conclusion

It has previously been demonstrated that that a greater $\dot{V}$O_2max_, higher *F* and lower Cr all contribute to successful endurance performance (Gimenez et al. 2013; Joyner and Coyle 2008; Lazzer et al. 2012; Millet et al. 2011; Saunders et al. 2004). However, in this treadmill ultramarathon, $\dot{V}$O_2max_ did not contribute to overall performance, with *F* and Cr explaining 61% of variance in finish time. In this participant cohort, $\dot{V}$O_2max_ is less of a performance predictor than *F* and Cr as event distance increases. Those able to maintain a higher *F* while adopting strategies to minimise an increase in Cr may be best placed to maximise ultramarathon performance from a physiological standpoint. The small sample size, homogeneity and relatively high training status of the cohort along with a large number of other potential performance determinates (lab versus field study, nutritional and psychological factors) that may contribute to overall ultramarathon performance (Howe et al. 2019)_,_ mean that further research needs to be carefully designed (Vernillo et al. 2017) to adequately measure and establish as many key performance determinants as possible. This would then enable a better understanding of the limiting factors of extreme human performance.

## Data Availability

The datasets generated during and/or analysed during the current study are available from the corresponding author on reasonable request.

## Abbreviations

ANOVA: Analysis of variance
BMI: Body mass index
CHO: Carbohydrate
Cr: Energy cost of running
*F*: Fractional utilisation of $\dot{V}$O_2max_
HR: Heart rate
HR_max_: Maximum recorded heart rate
RER: Respiratory exchange ratio
RPE: Rating of perceived exertion
RMR: Resting metabolic rate
SL: Stride length
SPM: Strides per minute
$\dot{V}$CO_2_: Carbon dioxide production
$$\dot{V}$$_E_: Pulmonary ventilation
$$\dot{V}$$O_2_: Oxygen uptake
$\dot{V}$O_2max_: Maximal oxygen uptake capacity
${\rm{V}}_{\dot{V}{\text{O}}_{2\max }}$: Maximum treadmill speed obtained at $\dot{V}$O_2max_
